# Integration of QSAR and SAR methods for the mechanistic interpretation of predictive models for carcinogenicity

**DOI:** 10.5936/csbj.201207003

**Published:** 2012-07-01

**Authors:** Natalja Fjodorova, Marjana Novič

**Affiliations:** a National Institute of Chemistry, Hajdrihova 19, SI-1001 Ljubljana, Slovenia

**Keywords:** Counter propagation artificial neural network, mechanistic interpretation of model, carcinogenicity, (quantitative) structure-activity relationships, structural alerts, Toxtree application, Dragon descriptors, Kohonen maps, non-congeneric chemicals

## Abstract

The knowledge-based Toxtree expert system (SAR approach) was integrated with the statistically based counter propagation artificial neural network (CP ANN) model (QSAR approach) to contribute to a better mechanistic understanding of a carcinogenicity model for non-congeneric chemicals using Dragon descriptors and carcinogenic potency for rats as a response. The transparency of the CP ANN algorithm was demonstrated using intrinsic mapping technique specifically Kohonen maps. Chemical structures were represented by Dragon descriptors that express the structural and electronic features of molecules such as their shape and electronic surrounding related to reactivity of molecules. It was illustrated how the descriptors are correlated with particular structural alerts (SAs) for carcinogenicity with recognized mechanistic link to carcinogenic activity. Moreover, the Kohonen mapping technique enables one to examine the separation of carcinogens and non-carcinogens (for rats) within a family of chemicals with a particular SA for carcinogenicity. The mechanistic interpretation of models is important for the evaluation of safety of chemicals.

## Introduction

Carcinogenicity is among the toxicological endpoints that pose the highest public concern. The standard bioassays in rodents used to assess the carcinogenic potency of chemicals are time-consuming, costly and require the sacrifice of a large number of animals. Cancer bioassays should be reduced according to the EU regulation REACH (Registration, Evaluation, Authorisation and restriction of Chemicals) [1], while the Seventh Amendment to the EU cosmetics directive will ban the bioassay for cosmetic ingredients from 2013 [2].

For these reasons, there is a need for alternative methods for carcinogenicity testing. Quantitative structure activity relationship (QSAR) methods can contribute to reduction of the animal studies. To be accepted for regulatory use, the QSAR model should follow the five OECD principles [3]. The fifth principle is that the models should have a mechanistic interpretation, if possible. The goal of mechanistic interpretation of QSAR models is to find relationship between descriptors and the endpoint and to incorporate mechanistic understanding and/or biological information.

It is a challenge to represent a mechanistic interpretation for models for prediction of carcinogenic potency for different classes of chemicals (so called non-congeneric chemicals) as they are comprised of a wide diversity of molecular structures related to variety of biological mechanisms.

Both the statistically based and knowledge-based methods are used in carcinogenicity models for prediction of non-congeneric chemicals [4–7]. The statistically-based methods (MultiCASE, Leadscope, TOPKAT, LAZAR and CAESAR systems) rely on techniques such as multivariate analysis, rule-induction, artificial intelligence, cluster analysis, pattern recognition, etc.). They deal with limited or no prior chemical or biological classification according to mechanism of carcinogenicity [8]. The knowledge-based (or rule-based) methods (HazardExpert, OncoLogic, Toxtree, and DEREK systems) include toxicological knowledge, expert judgment and fuzzy logic taking into consideration toxicokinetics, toxicodynamics and metabolism related to processes with cellular macromolecules or receptors. Each of the above mentioned approaches has the potentials and limitations described in the literature [7]. The knowledge-based approaches provide opportunity to gain insight into the mechanism underlying the carcinogenicity. The main advantage of statistically-based models is higher accuracy of prediction.

In this study we have combined QSAR (statistically-based) with SAR (knowledge-based) approaches. The QSAR model provided information about an association between chemical features (expressed as chemical descriptors) and the endpoint being predicted (carcinogenicity in our case) while knowledge-based Toxtree expert system [9] was employed as a supporting tool in interpretation of obtained results in terms of possible mechanism of carcinogenic activity of studied chemicals (encoded in the carcinogenic SAs). The mechanistic basis of the QSAR model was determined *a posteriori* (after the modelling), by interpretation of the final set of training structures and descriptors belonging to topological, electro-topological, and hydrogen bonding descriptors, which express different aspects of shape and size of molecules, contain encoded information about electronic interactions of the atoms and comprise features of electrostatic interaction between molecules.

We have considered counter propagation artificial neural network (CP ANN) model for prediction of carcinogenicity containing twelve Dragon descriptors which from statistical point of view was correlated to carcinogenicity and showed good recall ability and acceptable accuracy of prediction (69%) as was reported in the article [10]. Then we implemented selected descriptors correlated with carcinogenicity for prediction of SAs for carcinogenicity. The inherent to CP ANN mapping technique (Kohonen maps) was applied to see the distribution of chemicals, individual descriptors (in weight level maps), carcinogenic potency (Yes/No) and SAs for carcinogenicity in the same 2D space. The integration of the CP ANN mapping technique with the decision tree based Toxtree module for carcinogenicity enables to get mechanistic interpretation of a CP ANN QSAR model. The correlation between statistically selected descriptors and the carcinogenic potency as well as the possible mechanism of carcinogenic action (encoded in SAs for carcinogenicity) was studied in this paper.

## Data and Methods

### The dataset

The 805 chemicals extracted from initial dataset of 1481 chemicals (taken from Distributed Structure-Searchable Toxicity (DSSTox) Public Database Network http://www.epa.gov/ncct/dsstox/sdf_cpdbas.html) were used for modelling. The carcinogenic potency for rats was selected as a response (see article [10]). The information about structural alerts for carcinogenicity and type of alert (genotoxic alert (GA), non-genotoxic (nGA) alert or no alert (NA)) for each chemical in the dataset was taken out from the Toxtree expert system.

**Figure 1:**
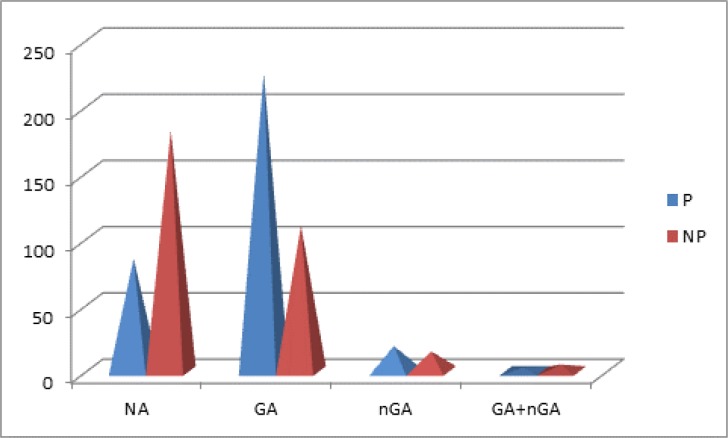
**The proportion of P and NP chemicals for rats for chemicals with GA, nGA, (GA+nGA) and NA**. Abbreviations: P- positive (carcinogen); NP- non positive (non-carcinogen); NA- no alert; GA- genotoxic alert; nGA- non genotoxic alert.

The diversity of dataset with indication of proportion of P- positive (carcinogen) and NP- non positive (non-carcinogen) chemicals for rats for chemicals with NA, GA, nGA is presented in Figure 1 and in Table S1 in Supplement Material section.

One should keep in mind that carcinogenic potency of chemicals in Toxtree knowledge-based system is based on observations in humans collected through epidemiological studies and on studies in animals while in CP ANN model we considered carcinogenic potency for rats.

The list of 33 SAs for carcinogenicity is reported by Benigni and Bossa [9] in the Toxtree rulebase. Carcinogenic SAs are functional groups or molecular substructures that were mechanistically and/or statistically associated with induction of cancer. In a broad sense the set of chemicals characterised by the same SA could compose a family of compounds with the same mechanism of action. A recent review [11] represents the information about chemical classes with recognized mechanistic link to carcinogenicity, coded as SA in the Toxtree 2.1.0 software. The list of SAs used in CP ANN model with indication of the number of chemicals in the dataset corresponding to particular SA is represented in Table 1. This table also contains the number of carcinogens (P) and non-carcinogens (NP) for each group of chemicals with a particular SA.

**Table:** **Table 1**. The list of structural alerts (SAs) used in CPANN model with indication number of carcinogens (P) and non-carcinogens (NP)

Structural Alert (SA)	Number of chemicals in datase with SA	P	NP
SA_21: alkyl and aryl N-nitroso groups	107	88	19
SA_27: Nitro-aromatic	75	48	27
SA_28: primary aromatic amine, hydroxyl amine and its derived esters	52	41	11
SA_8: Aliphatic halogens	47	25	22
SA_13: Hydrazine	32	19	13
SA_7:Epoxides and aziridines	22	13	9
SA_12: Quinones	22	10	12
SA_17: Thiocarbonyl	18	10	8
SA_28ter: aromatic N-acyl amine	17	11	6
SA_31a: Halogenated benzene	16	7	9
SA_18: Polycyclic Aromatic Hydrocarbons	12	9	3
SA_20: (Poly) Halogenated Cycloalkanes	9	5	4
SA_30: Coumarins and Furocoumarins	8	3	5
SA_5: S or N mustard	7	6	1
SA_14: Aliphatic azo and azoxy	7	6	1
SA_28bis: Aromatic mono- and dialkylamine	7	7	0
SA_31b: Halogenated PAH	7	2	5
SA_4: Monohaloalkene	5	5	0
SA_16: alkyl carbamate and thiocarbamate	5	5	0
SA_19: Heterocyclic Polycyclic Aromatic Hydrocarbons	5	4	1
SA_2: alkyl (C < 5) or benzyl ester of sulphonic or phosphonic acid	4	3	1
SA_11: Simple aldehyde	4	2	2
SA_15:: isocyanate and isothiocyanategroups	4	2	2
SA_25: aromatic nitroso group	4	4	0
SA_29: Aromatic diazo	4	4	0
SA_22: azide and triazene groups	3	2	1
SA_3: N-methylol derivatives	2	1	1
SA_31c: Halogenated dibenzodioxins	2	1	1
SA_9: Alkyl nitrite	1	1	0
SA_23: aliphatic N-nitro group	1	1	0
SA_24: a, b unsaturated aliphatic alkoxy group	1	1	0
SA_26: aromatic ring N-oxide	1	1	0

### Dragon descriptors

The DRAGON professional 5.4 program [12] has been employed for the calculation of 835 Dragon descriptors. Different descriptors represent different ways or perspectives to view a molecule, taking into account the mono-dimensional (e.g. the simple counts of atoms and groups), bi-dimensional (e.g. the topological graph) or three-dimensional (e.g. the minimum energy conformation) features. Thus, descriptors express different aspects of the shape and size of molecules, encode information about topological environment and electronic interactions of the atom and reflect the electrostatic interaction between molecules. The “Handbook of Molecular Descriptors” by Todeschini and Consonni [13] provides an encyclopaedic reference to molecular descriptors that are suitable for (Q)SAR studies.

The subset of twelve Dragon descriptors was identified using a statistical analysis (cross correlation matrix, multicolinearity and fisher ratio techniques). These Dragon descriptors maximally explain the variance in observed carcinogenic potency (property or activity of interest). Twelve Dragon descriptors selected for modeling are represented in Table 2.

**Table:** **Table 2**. Dragon descriptors selected for modeling.

Descriptor's sign	Symbol	Definition	Description
D1	PW5	path/walk 5 - Randic shape index	Topological descriptors (Molecular descriptors obtained from molecular graph (usually H-depleted), i.e. 2D-descriptors conformationally independent)
D2	D/Dr06	distance/detour ring index of order 6	Topological descriptors
D3	MATS2p	Moran autocorrelation - lag 2 / weighted by atomic polarizabilities	2D autocorrelations (Molecular descriptors calculated from molecular graph by summing the products of atom weights of the terminal atoms of all the paths of the considered path length (the lag). 2D autocorrelations by Moreau-Broto (ATS), Moran (MATS) and Geary (GATS) algorithms are calculated from lag 1 to lag 8 for 4 different weighting schemes)
D4	EEig10x	Eigenvalue 10 from edge adj. matrix weighted by edge degrees	Edge adjacency indices (Topological molecular descriptors derived from the edge adjacency matrix, which encodes the connectivity between graph edges)
D5	ESpm11x	Spectral moment 11 from edge adj. matrix weighted by edge degrees	Edge adjacency indices
D6	ESpm09d	Spectral moment 09 from edge adj. matrix weighted by dipole moments	Edge adjacency indices
D7	GGI2	topological charge index of order 2	Topological charge indices (First 10 eigenvalues (absolute values) obtained from a corrected adjacency matrix)
D8	JGI6	mean topological charge index of order6	Topological charge indices
D9	nRNNOx	number of N-nitroso groups (aliphatic)	Functional group counts (Molecular descriptors based on the counting of chemical functional groups_
D10	nPO4	number of phosphates/thiophosphates	Functional group counts
D11	N-067	Al2-NH	Atom-centred fragments (Molecular descriptors based on the counting of 120 atom-cantered fragments, as defined by Ghose-Crippen)
D12	N-078	Ar-N=X / X-N=X	Atom-centred fragments

Hereby, the molecular descriptors provide the information to generate the mechanistic interpretation of the underlying structure-activity or property relationship because they represent the relevant features of molecular structure that affect the observed properties (carcinogenicity) of a studied molecule.

### CPANN algorithm

The CPANN method was used in modelling, it belongs to self organizing map technique that is often used to analyse the data in multi-dimensional space. The basis of this technique is a non-linear projection from multi-dimensional space onto a two-dimensional map. The topology preserving projection is achieved via non-linear algorithm known as training. The fundamental property of the trained network is close vicinity of similar objects. Therefore, it is expected that chemicals with similar structure will form the clusters, which is the case of examination.

**Figure 2:**
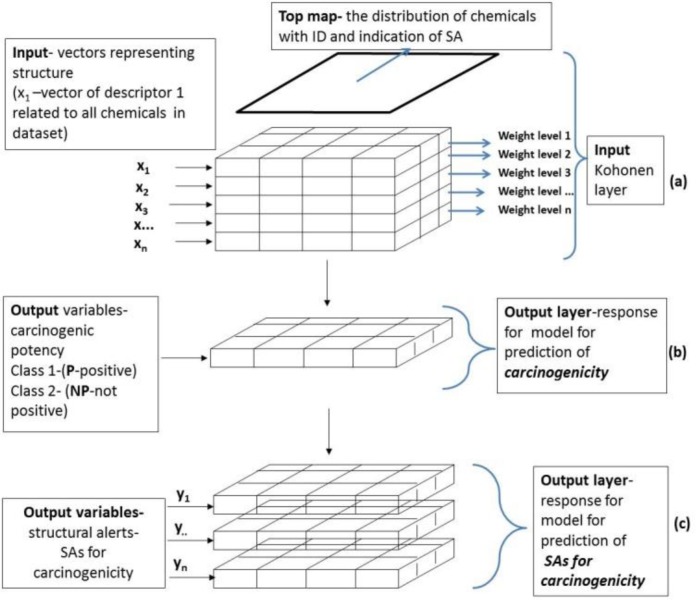
**The architecture of CPANN**. (**a**) input Kohonen layer; (**b**) output layer for model for prediction of *carcinogenicity*; (**c**) output layer for model for prediction of *SAs for carcinogenicity*

The architecture of CPANN is shown in Figure 2. The network constructed of neurons has two layers: input layer (Kohonen layer) containing encoded information of structure expressed as descriptors values and output layer (response). Both layers of neurons are placed exactly one above the other and the output layer has exactly the same layout of neurons as the input one [14]. The input layer has a number of levels (weights of the input neurons corresponding to the number of descriptors, i.e. the dimension of input vector X), while the output layer has as many levels as the target vectors have responses.

Kohonen maps enable visualisation of the distribution of chemicals (in the top map) and distribution of descriptors values (in weight levels maps). CP ANN, in turn, is a generalization of self organizing map. Additionally, it takes into account the property (output) values [15, 16] and is encompassed in the output layer. The learning in the input layer in the CPANN is the same as in Kohonen neural network, i.e., the similarity among input variables determines the arrangement of objects in the input layer map. When the arrangement is set the positions of objects are projected to the output where the weights are modified in a way that the weights on projected positions are getting similar to the values of corresponding objects.

The two kinds of models have been examined in the study: the model for prediction of carcinogenic class (model_cancer_*class*) and model for prediction of SAs for carcinogenicity (model_cancer_*SA*). It should be noted that input Kohonen layer is the same for both models while responses are different (see Figure 2).

In Figure 2 the inputs x_1_, x_2_, x_3,_..., x_n_ are vector components representing chemical structure which corresponds to descriptors calculated for all chemicals used in training dataset. In the other words, x_1i,_ x_2i_, x_3i,_..., x_ni_ can be represented as a matrix of descriptor 1, 2, 3..., n values for all of 644 chemicals (i=1,…, 644) in training dataset, respectively. The distribution of chemicals and their clusters in 2D space is examined in the Kohonen top map. Weight levels 1, 2, 3,..., n are the maps with distribution of particular descriptors 1, 2, 3,..., n, correspondingly. Output variables are expressed in the output layer as a carcinogenicity class (class 2 was marked as carcinogen and class 1 as non-carcinogen) in models for prediction of carcinogenicity and as particular SAs for carcinogenicity in the models for prediction of SAs.

In the study the following 2D maps were used to analyse the similarities in non-congeneric set of substances: Kohonen top map (distribution of chemicals), weight levels maps (distribution of individual descriptors) (Figure 2(a)), corresponding response surface output layer maps with distribution of carcinogenicity class (carcinogens/non carcinogens) in model for prediction of carcinogenicity (Figure 2(b)), and distribution of SAs for carcinogenicity in model for prediction of SAs (Figure 2(c)).

## Results and Discussions

### CP ANN model for prediction of carcinogenic class

#### Special features of the CP ANN model for prediction of carcinogenic class

In the first part of study we represented the CP ANN model for prediction of carcinogenic class (carcinogen (2) and non-carcinogen (1)) (model_cancer_*class*). We examined model based on twelve Dragon descriptors with 35x35 dimensional artificial neural network (ANN) and trained for 200 epochs (see paper [10]).

For the model validity a wide series of statistical checks have been used. Models yielded the accuracy of training set (644compounds) equal to 89%; the accuracy of the test set (161compounds) was 69%. The accuracy of the leave 20% out cross validation for the training set was equal to 62%. To verify if the models perform correctly on new compounds the external validation was carried out. The external test set was composed of 738 compounds. We obtained accuracy of external validation equal to 60.0%.

The relationships between carcinogenic class, structure of chemicals and applied descriptors were in focus of our investigations.

Because of the diversity of the molecules studied in this work, the carcinogenic property of the compounds is related to the molecular structure in a complex way. The descriptors used in the study encode different aspects of the molecular structure. We have used the CP ANN and combined the mapping capability of a Kohonen network with a supervised learning strategy. We examined the pattern levels in the weights of trained network which provide the researcher with a deeper knowledge about mechanistic background related to the effect of individual variables visualized and estimated from the formed clusters.

The present paper resumes and upgrades the mechanistic interpretation of model using twelve Dragon descriptors reported in the paper [10]. We have considered the top map of Kohonen layer (with distribution of chemicals), weight level maps of descriptors variables and output layer corresponding to the carcinogenic class to show connection between the structure features of chemicals, individual descriptors and corresponding carcinogenic class.

#### The correlation between carcinogenic potency, nitroso compounds and Dragon descriptors

**Figure 3:**
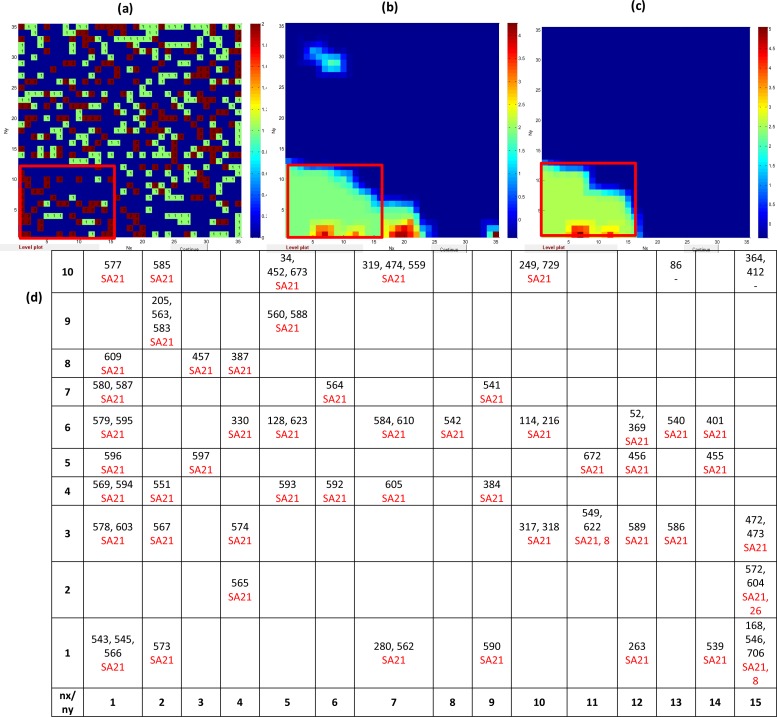
(**a**) The output layer of model for prediction of *carcinogenicity* with distribution of carcinogens (2) and non-carcinogens (1); (**b**) weight map of Dragon descriptor D12 (*N-078-* Ar-N=X / X-N=X); (**c**) weight map of Dragon descriptor D9 (*nRNNOx-* number of N-nitroso groups (aliphatic)); (**d**) a fragment of the bottom left section of the top map (35x35) with coordinates (n_x_ from 1 to 15 and n_y_ from 1 to 10) labelled with ID of chemicals occupying individual neurons with indication of SA for carcinogenicity. **Note:** Fragment (**d**) corresponds to clusters marked as red rectangle in (**a**), (**b**) and (**c**).

We investigated the distribution of carcinogens and non-carcinogens in the output layer of model_cancer_*class* and have found in the left bottom section of the map (Figure 3a) an area populated with a majority of carcinogens (2). Firstly, we considered an output layer of the model which represents the map 35*35 in the x and y direction. Green small squares (1) in the output layer (Figure 3a) are non-carcinogens, while brown small squares (2) are carcinogens.

Figure 3d shows the fragment of the bottom left section of this map 35x35 of model_cancer_*class* (see red rectangle) with coordinates (n_x_ from 1 to 15 and n_y_ from 1 to 10) labelled with ID of chemicals occupying individual neurons with indication of SAs for carcinogenicity. Additionally, the weight maps of Dragon descriptors and D12 (*N-078- Ar-N=X / X-N=X*) and D9 (*nRNNOx- Number of N-nitroso groups (aliphatic)*) are shown in Figure 3b and Figure 3c, respectively. The weight maps illustrate the distribution of values of particular descriptor (D12 or D9 in our case) over the 35*35 map. The red, yellow and then light green color corresponds to the highest values, while dark blue relates to the smallest values.

The majority of chemicals located in selected area (red rectangle) contains *SA21* and are carcinogens. Thus, Figure 3 demonstrates the correlation between carcinogenic potency related to chemicals contained the structural alert for carcinogenicity *SA21* (*alkyl and aryl N-nitroso groups*) and consequently with the Dragon descriptors D12 (*N-078- Ar-N=X / X-N=X*) and D9 (*nRNNOx- Number of N-nitroso groups (aliphatic)*).

Indeed, the descriptors identify the certain structural features or particularities. Descriptor D9 corresponds to *Functional group counts* (number of N-nitroso groups (aliphatic) while D12 relates to *Atom-centred fragments* (Ar-N=X / X-N=X).

Thus, we have found the relationship between descriptors containing features for nitro compounds that gave ability to neural network to organize those families of chemicals in topologically near locations (neurons). The majority of chemicals from this class are carcinogens, i.e. possessed the same biological activity. Obviously, the nitro SAs are important for carcinogenic activity which is in good agreement with the selection of Dragon D9 and D12 descriptors that resulted from our modelling methodology (see article [10]).

#### The study of influential zones of Dragon descriptors

The following part of our study was dedicated to research of influential zones of descriptors (areas with the largest values) and their correlation with structure of chemicals located in those areas. Analysing the individual descriptors layers in Self-Organizing Maps one recognized the importance and role of individual descriptors in a studied model. The results of our investigations are represented in the supplementary material section in Tables S1- S9.

**Figure 4:**
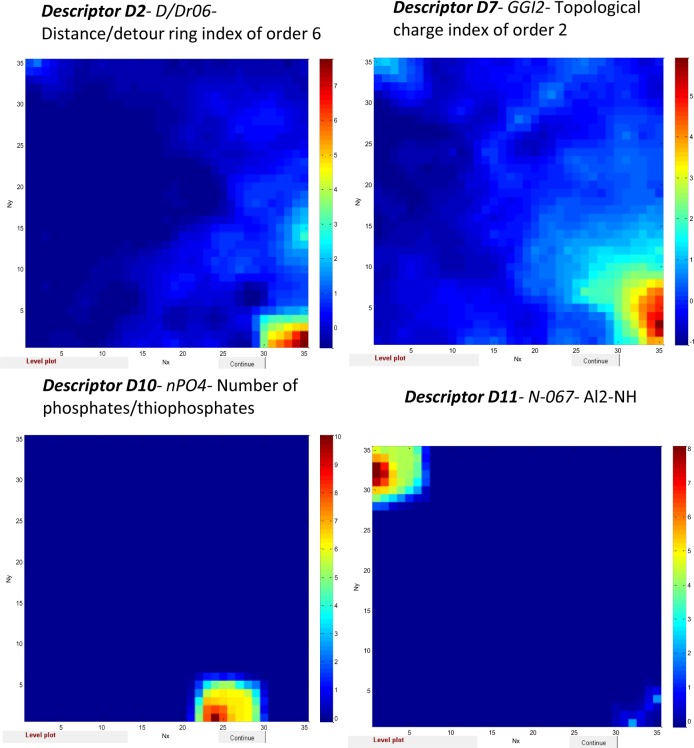
Weight maps of Dragon descriptors D2, D7, D10 and D11 with small influential area related to non-carcinogens.

After careful consideration of data represented in Tables S1- S9 we have found that influential zones of some of Dragon descriptors like D2 (*D/Dr06-* distance/detour ring index of order 6) (Figure S2), D7 (*GGI2-* topological charge index of order 2) (Figure S4), D10 (*nPO4-* Number of phosphates/thiophosphates) (Figure S7), D11 (*N-067- Al2-NH*) (Figure S8) have small limited area (see Figure 4) corresponding to non-carcinogens. The influential areas of descriptors D2, D7, D10 and D11 do not match up with influential areas of descriptors D9 and D12 (see Figure 3) related to location of carcinogens. Possibly the descriptors D2, D7, D10 and D11 might have features explained the non-carcinogenic properties while descriptors like D9 and D12 correspond to carcinogenic property of compounds.

**Figure 5:**
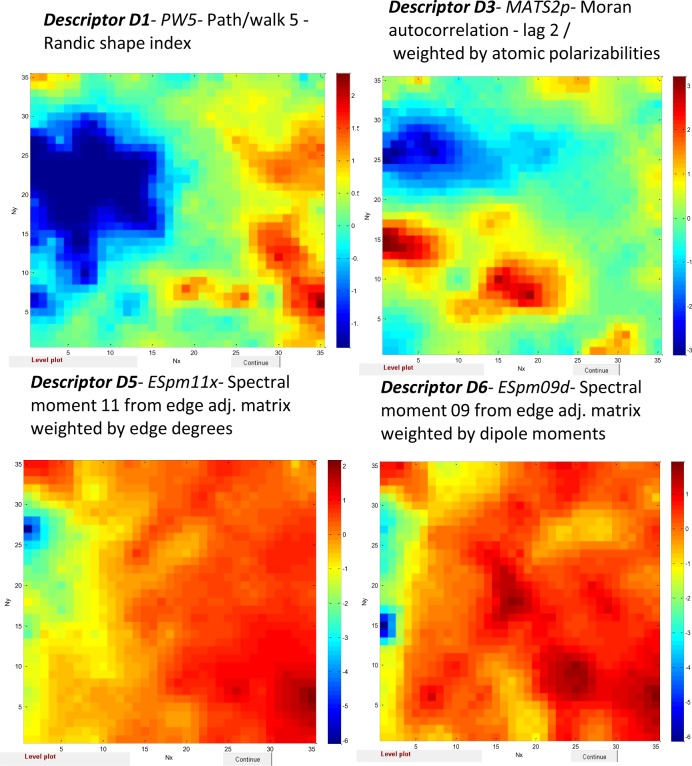
Weight maps of Dragon descriptors D1, D3, D5 and D6 with wide influential zone over the whole map.

In contrast, the Dragon descriptors like D1 (*PW5- Path/walk 5 -* Randic shape index) (Figure S1), D3 (*MATS2p*- Moran autocorrelation - lag 2 / weighted by atomic polarizabilities) (Figure S3), D5 (*ESpm11x-* Spectral moment 11 from edge adj. matrix weighted by edge degrees) and D6 (*ESpm09d-* Spectral moment 09 from edge adj. matrix weighted by dipole moments) (Figure S5) have influential zone spread over whole map (see Figure 5). This phenomenon probably is the evidence that these descriptors have features that affect the majority of chemicals in the dataset and their properties.

The Dragon descriptors D4 and D7 (Figure S4), D5 and D6 (Figure S5) and D12 and D9 (Figure S9) have influential zones in the similar locations.

Several descriptors were found to have overlapping influential zones for the same chemicals. Figure S4 demonstrates this phenomenon. Beta-Cyclodextrin; (CASRN 7585-39-9) and Vinblastine; (CASRN 865-21-4) were found in the influential zones of Dragon descriptors D4 and D7 (Figure S4). Beta-Cyclodextrin; (CASRN 7585-39-9) is also located in the influential zone of Dragon descriptor D2 (Figure S2). It means that descriptors selected for characterization of carcinogenicity have similar features. In the second example two non-genotoxic halogenated cycloalkanes chemicals with *SA_20* (Mirex, photo- (*CASRN 39801-14-4*) and Chlordecone (kepone) (*CASRN 143-50-0*)) were found in influential zones of Dragon descriptors D1 (Figure S1), D5 and D6 (Figure S5).

### CP ANN model for prediction of SAs for carcinogenicity

#### How CP ANN algorithm create clusters of chemicals containing particular SA for carcinogenicity

In the first part of the study we described the CP ANN model for prediction of *carcinogenic class* (model_cancer_*class*). Model for prediction of *SA for carcinogenicity* (model_cancer_*SA*) represented in the second part of the study was based on the same input data (the numeric representation of the twelve Dragon descriptors). 35x35 dimensional ANN was trained for 200 epochs.

**Figure 6:**
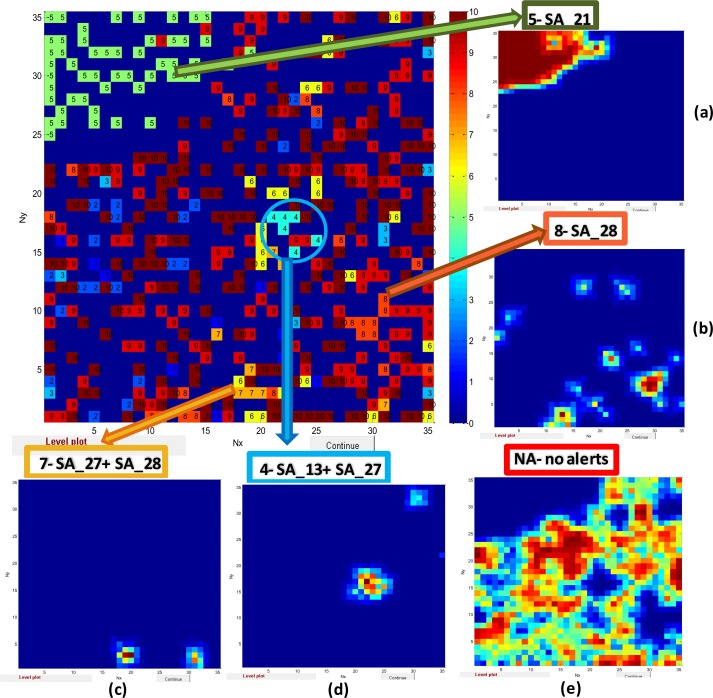
The output layer of model for prediction of *SAs for carcinogenicity* with the distribution of the largest selected families of chemicals containing appropriate SAs (from 1 to 10*) complemented with the following weight maps demonstrating distribution of chemicals containing: (**a**) *SA_21*- (**5**); (**b**) *SA_28*- (8); (**c**) (*SA_27+SA_28)*- (**7**); (**d**) (*SA_13+SA_27)*- (4); (**e**) *NA* (no alerts)- (10). **Notes:** *The numbers (1-10) in top map relate to chemicals containing the following SAs: **1**- *SA7*, **2**-*SA8*, **3**-*SA13*, **4**-(*SA13+SA27)*, **5**-*SA21*, **6**-*SA27*, **7**-(*SA27+SA28)*, **8**-*SA28*, **9**- *SA_X* (X-others SA), **10**-*NA*

Firstly, we considered an output layer of the model which represents the map 35*35 with destribution chemicals containing a particular SA. The numbers (1-10) in top map relate to chemicals containing the following SAs: 1- *SA7*, 2-*SA8*, 3-*SA13*, 4-(*SA13+SA27)*, 5-*SA21*, 6-*SA27*, 7-(*SA27+SA28)*, 8-*SA28*, 9- *SA_X* (X-others SA), 10-*NA*. SAs are marked with different colour. For example, 5-*SA21* corresponds to green colour, 8-*SA28* corresponds to orange colour, and 6-*SA27* corresponds to yellow colour and so on (see Figure 6).

In this study the largest groups of chemicals with the following SAs were considered: 5- *SA_21* (alkyl and aryl N-nitroso groups) (107chemicals); 6- *SA_27* (Nitro-aromatic) (75 chemicals); 8- *SA_28* (primary aromatic amine, hydroxyl amine and its derived esters) (52 chemicals); 2- *SA_8* (aliphatic halogens) (47 chemicals); 3- *SA_13* (hydrazine) (32 chemicals); 1- *SA_7* (epoxides and aziridines) (22chemicals). We also considered chemicals containing two different SAs in one molecule: 4- (*SA_13*+*SA_27*) (12 chemicals) and 7- (*SA27*+*SA_28*) (14chemicals). We used in study 334 chemicals which have no carcinogenic alerts (10- *NA*-no alert). Others chemicals were marked as 9- *SA_X* contain others (X) SAs. Table 1 represents the list of structure alerts (SAs) for carcinogenicity used in the modelling with indication of the total number of chemicals in the dataset containing particular SAs and number of carcinogens (P) and non-carcinogens (NP) for each group of chemicals with particular SA.

The Kohonen map enables to get clusters of congeneric substances as well as to get insight within congeneric sets of chemicals and to determine similarities or dissimilarities within groups of chemicals characterised on the basis of particular carcinogenic SA. The weight levels maps with distribution of the chemicals containing the particular SAs (5-*SA_21*; SA 8- *SA_28*; 7-(*SA_27+SA_28*)*;* 4- (*SA13 +SA27*) and NA (no alert)) are shown in Figure 6(a), (b), (c), (d), and (e), correspondingly. The weight maps illustrate the distribution of values of particular SA over the 35*35 map. The highest values correspond to red, yellow and then to light green color, while dark blue relate to the smallest values.

We have noticed that the following groups of chemicals generate one cluster: 5-*SA_21* (nitro compounds) (Figure 6a) and 4- (*SA13 +SA27*) (hydrazine and nitro-aromatic) (Figure 6d). Two clusters are visible in the case of 7-(*SA_27+SA_28*) (nitro-aromatic, primary aromatic amines) (Figure 6c), while others groups of chemicals marked as 1, 2, 3, 6, 8, 9 and 10 are scattered in the whole map. The chemicals with SA 8- *SA_28* (Figure 6b) have several clusters spread over the map.

As the distribution of chemicals in the Kohonen map caused by similarity in their activity we have considered the mechanism of action for groups of chemicals marked as 5, 4, 7 and 8 (see Figure 6).

#### Mechanism of action intrinsic to studied groups of chemicals

Thus, the N-Nitroso compounds (5) containing *SA_21* belongs to alkylating, indirect acting agent. The mechanism of action intrinsic to N-Nitroso compounds containing *SA_21* reported in papers [11, 17]. We can conclude that chemicals containing the *SA21* possess the similar activity. Indeed, N-nitrosamines and N-nitrosamides represent a well established class of chemical carcinogens as was reported in paper [11].

The cluster marked with number (4) corresponds to compounds containing two SAs: (*SA_27+SA_13*) (*nitro-aromatic* compounds and *hydrazines*- chemicals). The hydrazines (chemicals containing *SA13*) belong to alkylating, indirect acting agent while nitroaromatic compounds (containing *SA27*) belong to aminoaryl DNA-adducts forming, indirect acting agents. The structures of studied compounds (12 chemicals) are represented in Table S2 in Supplement material section. It should be noticed that only one chemical from this dataset is non-carcinogen (C.I. Pigment red 23 (CASRN 6471-49-4)). This compound has molecular weight (MW) equal to 486. The chemicals with very high MW and size have little chance to be absorbed in significant amounts and therefore are non-active. The rest of the chemicals are carcinogens and have MW between 200 and 300. The detailed explanation of mechanism of action intrinsic to chemicals contained *SA27* and *SA13* is reported in paper [11].

Thus, the Dragon descriptors represent such features that organize the group of chemicals (with *SA13+SA27*) in clear visible cluster in Kohonen map which is the evidence of similarity. Indeed, almost all chemicals (one exception) are active (carcinogens). Table S2 demonstrates that 12 chemicals presented in this list have similar structure and as a result possess the same activity.

The cluster marked with number (7) corresponds to compounds containing two SAs: SA_27: *Nitro-aromatic*+SA_28: *primary aromatic amine, hydroxyl amine and its derived esters* (14 chemicals) (Figure 6c). Both classes of chemicals belong to aminoaryl DNA-adducts forming, indirect acting agents. Clusters of chemicals containing those two alerts (*SA27+SA28*) (see Table S3) are placed in the closest neurons because of similarity due to similar structure and presence of the same groups responsible for the similar mode of action or biological activity. It is interesting that all chemicals in this cluster are positive by results of mutagenicity tests (Salmonella typhimurium TA98 strain).

The chemicals marked with number (8) correspond to compounds containing *SA28* (*primary aromatic amine, hydroxyl amine and its derived esters*). These 52 chemicals are spread over the Kohonen map due to big diversity of structures and presence of many others functional groups (see Table S4 in the Supplement Material Section).

As for distribution of chemicals marked as *NA* (no alert) one can see that chemicals are distributed over the map and difficult to separate individual clusters (see weight level map of *NA* in the Figure 6e).

#### How CP ANN algorithm separates carcinogens (P) and non-carcinogens (NP) inside group of chemicals containing a particular SA

**Figure 7:**
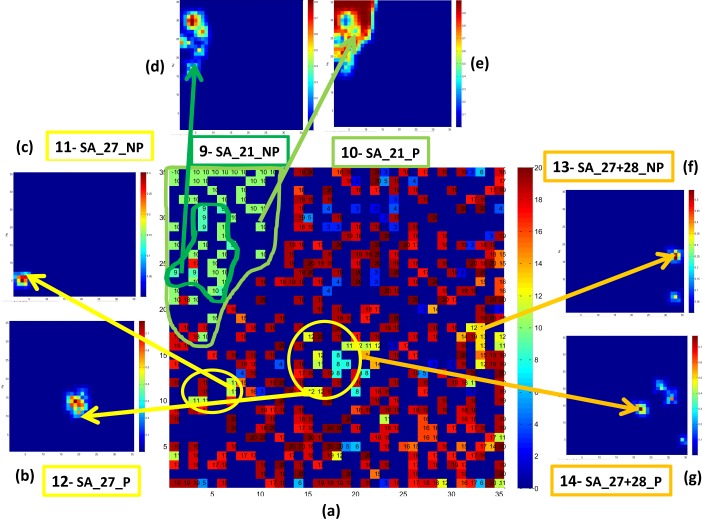
(**a**) the output layer of CP ANN model for prediction of *SAs for carcinogenicity* complemented with the weight maps illustrating the distribution of carcinogens (P) and non-carcinogens (NP) for chamicals containing the following individual SAs: SA_27 (**b**, **c**), SA_21 (**d**, **e**), and SA_27+28 (**f**, **g**).

The intrinsic to CP ANN Kohonen mapping technique enables to see the distribution of carcinogens (P) and non-carcinogens (NP) for rats inside different families of chemicals with particular SA for carcinogenicity. Figure 7 demonstrates the output layer of CP ANN model for prediction of *SAs for carcinogenicity* complemented with the weight maps illustrating the distribution of carcinogens (P) and non-carcinogens (NP) for chemicals containing the following individual SAs: SA_27 (b, c), SA_21 (d, e), and SA_27+SA_28 (f, g).

Location of P and NP chemicals differs in the Kohonen maps due to different activity of considered chemicals. It should be highlighted that carcinogenic potency of chemicals in Toxtree knowledge-based system is based on observations in humans collected through epidemiological studies and on studies in animals while in CP ANN model we considered carcinogenic potency for rats. This is why we used SA for carcinogenicity only for explanation of possible mechanism of carcinogenic activity in broad sense. Determination of the differences inside the congeneric chemicals (with particular SA for carcinogenicity) using Kohonen mapping is very important for evaluation of safety of chemicals.

## Conclusion

Mechanistic insight into CP ANN models was demonstrated using the inherent mapping technique (i.e. Kohonen maps) which enables the visualization of the following features in 2D space: the carcinogenic potency; the distribution of descriptors in individual layers; and the distribution of congeneric groups of chemicals with indication of specific carcinogenic SAs with indication of broad mechanisms of action.

We have examined statistically selected twelve Dragon descriptors which are correlated with carcinogenicity. These descriptors express structural and electronic features such as molecular shape (linear, branched, cyclic, and polycyclic), bond length, taking into account electronic surrounding of molecular.

It was shown the correlation between carcinogenic potency related to chemicals contained the structural alert for carcinogenicity *SA21* (*alkyl and aryl N-nitroso groups*) and consequently with the Dragon descriptors D12 (*N-078- Ar-N=X / X-N=X*) and D9 (*nRNNOx- Number of N-nitroso groups (aliphatic)*). Thus, the *functional group counts* descriptor D9 and *atom-centred fragments* descriptor D12 match up with structural alert for carcinogenicity *SA21-*(alkyl and aryl N-nitroso groups) and consequently correlated with carcinogenic activity of chemicals.

In turn, the *functional group counts* (fragment –based) descriptor like D10 (*nPO4- Number of phosphates/thiophosphates)* demonstrated the ability to characterize the lack of carcinogenicity (for example in case of phosphates/thiophosphates).

The influential zone of non-fragment count descriptors like D2 (*D/Dr06-* distance/detour ring index of order 6), D7 (*GGI2-* topological charge index of order 2), and D11 (*N-067- Al2-NH*) related to non-carcinogens. These descriptors might have features explained the non-carcinogenic properties. They match neither the SAs for carcinogenicity nor the location of carcinogens related to descriptors like D9 and D12 correlated with carcinogenicity.

The non-fragment descriptors like D1 (*PW5- Path/walk 5 -* Randic shape index), D3 (*MATS2p*- Moran autocorrelation - lag 2 / weighted by atomic polarizabilities), D5 (*ESpm11x-* Spectral moment 11 from edge adj. matrix weighted by edge degrees) and D6 (*ESpm09d-* Spectral moment 09 from edge adj. matrix weighted by dipole moments) have influential zone spread over the whole map. One would not expect to be able to locate specific regions where these descriptors are very important. These descriptors have features that affect the majority of chemicals and their properties in the dataset.

It was demonstrated how the Kohonen mapping technique enables to separate chemicals within a family of chemicals with particlular SA by their activity (by mechanism of action) as well as by their carcinogenic activity (carcinogens and non-carcinogens)

It was illustrated that inside a family of chemicals they can be spread over the Kohonen map and have several clusters due to big diversity of structures and presence of many others functional groups which causes the differences in their activity.

The QSAR and SAR approaches have been integrated to receive more comprehensive data for risk assessment.

## Supplementary Material

Integration of QSAR and SAR methods for the mechanistic interpretation of predictive models for carcinogenicity
