# Supplementary material for: Integration of QSAR and SAR methods for the mechanistic interpretation of predictive models for carcinogenicity
Source: Comput Struct Biotechnol J. 2012 Jul 1;1:e201207003. doi: 10.5936/csbj.201207003 (PMC3962111; doi:10.5936/csbj.201207003)
Supplement: Integration of QSAR and SAR methods for the mechanistic interpretation of predictive models for carcinogenicity [file CSBJ-1-e201207003_SM0001.pdf]

Supporting Information

Integration of QSAR and SAR methods for the mechanistic interpretation of predictive models for carcinogenicity

Natalja Fjodorova[1]*, Marjana Novič[1]

Addresses:
1 National Institute of Chemistry, Hajdrihova 19, SI-1001 Ljubljana, Slovenia

Emails:
Natalja Fjodorova* - natalja.fjodorova@ki.si;
tel.: +386 14760290; fax: +38614760300

Marjana Novič- marjana.novic@ki.si;

* Corresponding author

The captures of Figures

Figure S1. (**a**)- The output layer of model_cancer_***class*** with distribution of carcinogens (**2**) and non-carcinogens (**1**); (**b**)- The weight map corresponding to descriptor ***D1***- (*PW5- Path/walk 5* - Randic shape index); (**c**)- The name and structure of chemicals placed in the influential zone of descriptor ***D1*** with indication of position in Kohonen map.

Figure S2. (**a**)- The output layer of model_cancer_***class*** with distribution of carcinogens (**2**) and non-carcinogens (**1**); (**b**)- The weight map corresponding to descriptor ***D2***- (*D/Dr06*- Distance/detour ring index of order 6); (**c**)- The name and structure of chemicals placed in the influential zone of descriptor ***D2*** with indication of position in Kohonen map.

Figure S3. (**a**)- The output layer of model_cancer_***class*** with distribution of carcinogens (**2**) and non-carcinogens (**1**); (**b**)- The weight map corresponding to descriptor ***D3***- (*MATS2p*- Moran autocorrelation - lag 2 / weighted by atomic polarizabilities); (**c**)- The name and structure of chemicals placed in the influential zone of descriptor ***D3*** with indication of position in Kohonen map.

Figure S4. (**a**)- The output layer of model_cancer_***class*** with distribution of carcinogens (**2**) and non-carcinogens (**1**); (**b1, b2**)- The weight maps corresponding to descriptors ***D4*** and ***D7*** correspondingly; (**c**)- The name and structure of chemicals placed in the influential zone of descriptors ***D4*** and ***D7*** with indication of position in Kohonen map.

Figure S5. (**a**)- The output layer of model_cancer_***class*** with distribution of carcinogens (**2**) and non-carcinogens (**1**); (**b1, b2**)- The weight maps corresponding to descriptors ***D5*** and ***D6***, respectively; (**c**)- The name and structure of chemicals placed in the influential zone of descriptors ***D5*** and ***D6*** with indication of position in Kohonen map.

Figure S6. (**a**)- The output layer of model_cancer_***class*** with distribution of carcinogens (**2**) and non-carcinogens (**1**); (**b**)- The weight maps corresponding to descriptor ***D8*** (*JGI6*- Mean topological charge index of order6); (**c**)- The name and structure of chemicals placed in the influential zone of descriptor ***D8*** with indication of position in Kohonen map.

Figure S7. (**a**)- The output layer of model_cancer_***class*** with distribution of carcinogens (**2**) and non-carcinogens (**1**); (**b**)- The weight maps corresponding to descriptor ***D10 (*** *nPO4*- Number of

phosphates/thiophosphates); (**c**)- The name and structure of chemicals placed in the influential zone of descriptor ***D10*** with indication of position in Kohonen map.

Figure S8. (**a**)- The output layer of model_cancer_***class*** with distribution of carcinogens (**2**) and non-carcinogens (**1**); (**b**)- The weight maps corresponding to descriptor ***D11*** **(***N-067- Al2-NH*) ; (**c**)- The name and structure of chemicals placed in the influential zone of descriptor ***D11*** with indication of position in Kohonen map.

Figure S9. (**a**)- The output layer of model_cancer_***class*** with distribution of carcinogens (**2**) and non-carcinogens (**1**); (**b1, b2**)- The weight maps corresponding to descriptors ***D12*** and ***D9***, respectively; (**c**)- The name and structure of chemicals placed in the influential zone of descriptors ***D12*** and ***D9*** with indication of position in Kohonen map.

Notes:* Abbreviations in **Figures 1SI-9SI: GA- genotoxic alert; NA- no alert; P- positive (carcinogen); NP- non positive (non-carcinogen).

The captures of Tables_SI

Table S1 The diversity of dataset used in CP ANN model with indication of number of carcinogens (P) and non-carcinogens (NP) as well as number of chemicals without carcinogenic alert (NA), genotoxic alerts (GA) and non-genotoxic alert (nGA)

Table S2 Chemical structure of compounds contating (*SA13 + SA27*) (12 compounds)

Table S3 Chemical structure of compounds contating (*SA27_SA28*) (12 compounds)

Table S4 Chemical structure of compounds containing *SA_28* (52 compounds)

Dragon descriptor D1- *PW5*- Path/walk 5 - Randic shape index

(a) (b)

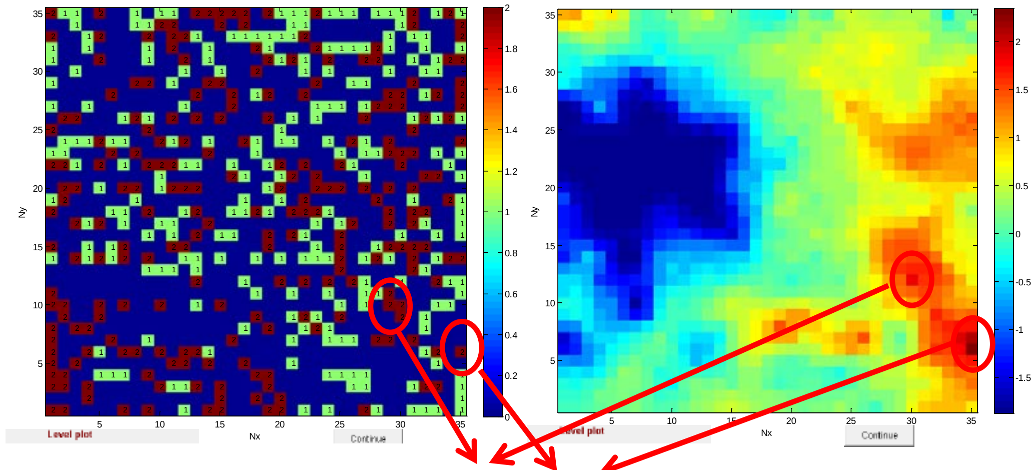

Position - n_x*n_y=29*10; 35*6

(c)

Position of n_x*n_y=29*10;
Decabromodiphenyl oxide;
(CASRN 1163-19-5);
nGA(SA_31b); P

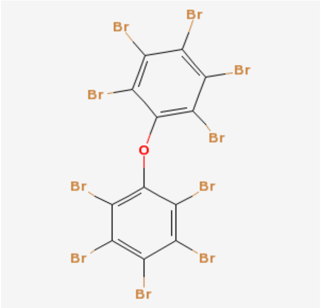

Position of n_x*n_y=35*6;
Mirex, photo-;
(*CASRN 39801-14-4*);
SA20; P

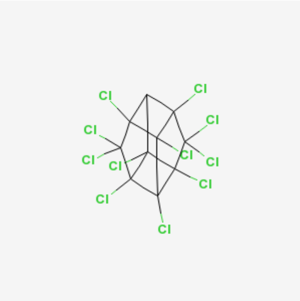

Position of n_x*n_y=35*6;
Chlordecone (kepone);
(*CASRN 143-50-0*);
SA20; P

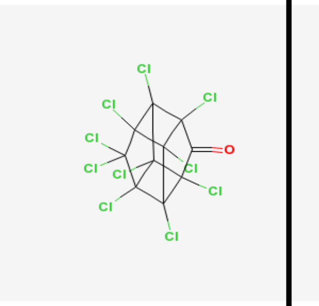

SA_31b: Halogenated PAH (nogenotoxic)
Ar----[Br,Cl,F,I]; where
Ar = naphthalene, biphenyl, diphenyl

SA_20: (Poly) Halogenated Cycloalkanes (nongenotoxic)
Any cycloalkane skeleton with three or more halogens directly bound to the same ring

Figure S1. (**a**)- The output layer of model_cancer_***class*** with distribution of carcinogens (**2**) and non-carcinogens (**1**); (**b**)- The weight map corresponding to descriptor ***D1***- (*PW5*- *Path/walk 5* - Randic shape index); (**c**)- The name and structure of chemicals placed in the influential zone of descriptor ***D1*** with indication of position in Kohonen map.

Dragon descriptor D2- *D/Dr06*- Distance/detour ring index of order 6

(a) **(b)**

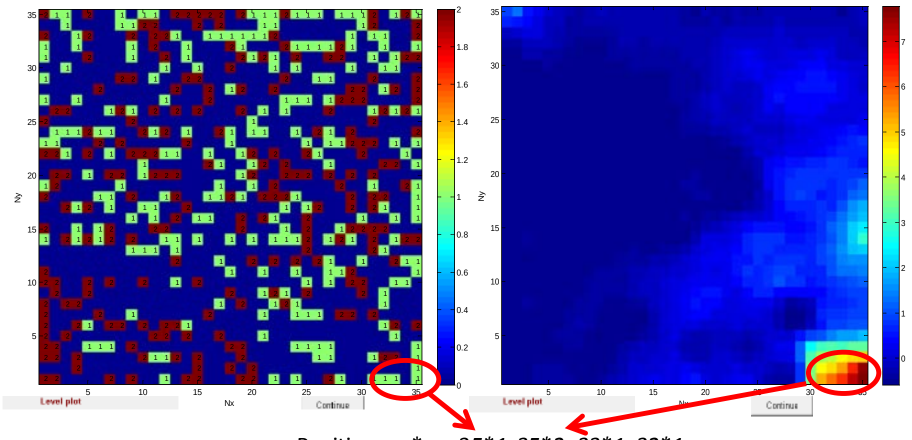

(c)

Position - n_x*n_y=*35*1; 35*2; 33*1; 32*1*

Position of n_x*n_y=*33*1*
Estradiol mustard;
(*CASRN 22966-79-6*);
SA5; NP in rat and P in mouse

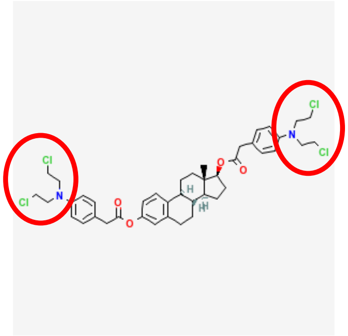

SA_5: S or N mustard
R = any atom/group

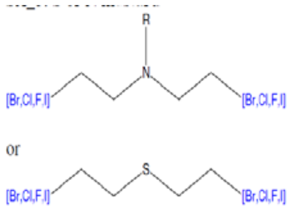

Position of n_x*n_y=*32*1;*
Azelnidipine:
(CASRN 123524-52-7);
SA27; NP in rat and mouse

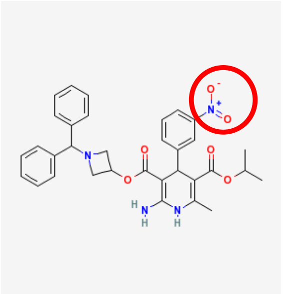

SA_27: Nitro-aromatic

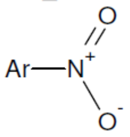

Ar = Any aromatic/heteroaromatic ring
• Chemicals with ortho-disubstitution, or with an ortho carboxylic acid substituent are excluded.
• Chemicals with a sulfonic acid group (-SO3H) on the same ring of the nitro group are excluded .

Position - n_x*n_y=*35*1;*
Diarylanilide yellow;
(*CASRN 6358-85-6*);
NA; NP in rat and mouse

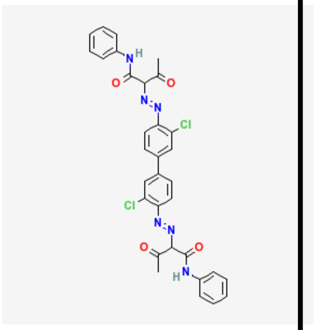

Position -n_x*n_y=*35*2;*
beta-Cyclodextrin;
(*CASRN 7585-39-9*);
NA; NP in rat and mouse

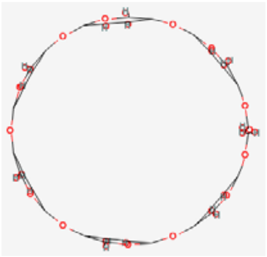

Figure S2. (**a**)- The output layer of model_cancer_***class*** with distribution of carcinogens (**2**) and non-carcinogens (**1**); (**b**)- The weight map corresponding to descriptor ***D2***- (*D/Dr06*- Distance/detour ring index of order 6); (**c**)- The name and structure of chemicals placed in the influential zone of descriptor ***D2*** with indication of position in Kohonen map.

Dragon descriptor D3- *MATS2p*- Moran autocorrelation - lag 2 / weighted by atomic polarizabilities

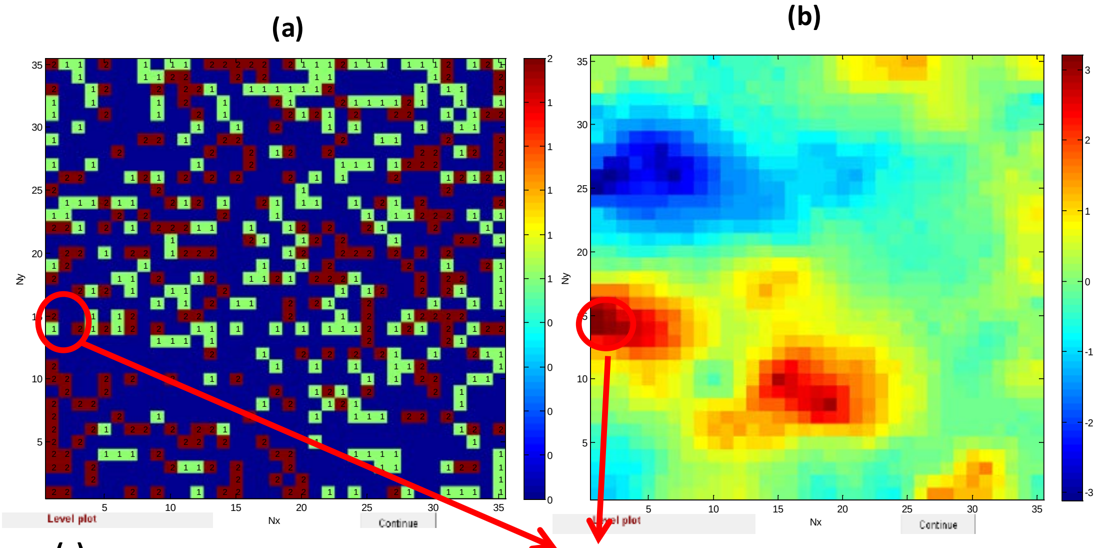

(c)

Position - n_x*n_y=3**14; 1*14; 1*15*

Position of nx*ny=1*15;
Benzene;
(*CASRN 71-43-2*);
NA; P

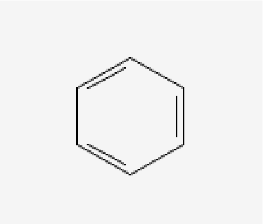

Position nx*ny=3*14;
Ethylbenzene;
(*CASRN 100-41-4*);
NA; P

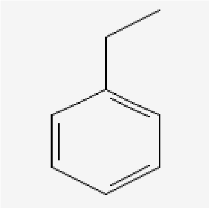

Position nx*ny=3*14;
Melamine;
(*CASRN 108-78-1*);
SA28; P

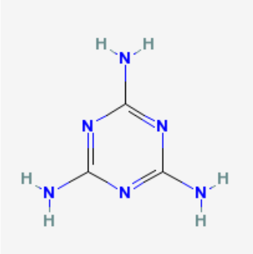

Position of nx*ny=1*15-
1,3-Butadiene;
(CASRN 106-99-0);
NA; P

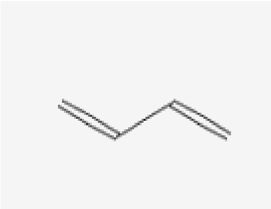

Position of nx*ny=1*14;
Propylene;
(*CASRN 115-07-1*);
NA; NP

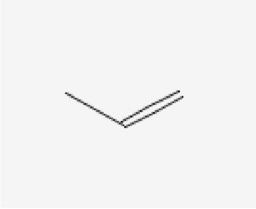

SA_28: primary aromatic amine, ...
Ar = Any aromatic/ heteroaromatic ring

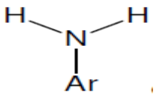

Figure S3. (**a**)- The output layer of model_cancer_***class*** with distribution of carcinogens (**2**) and non-carcinogens (**1**); (**b**)- The weight map corresponding to descriptor ***D3***- (*MATS2p*- Moran autocorrelation - lag 2 / weighted by atomic polarizabilities); (**c**)- The name and structure of chemicals placed in the influential zone of descriptor ***D3*** with indication of position in Kohonen map.

Dragon descriptor D4- *EEig10x*- Eigenvalue 10 from edge adj. matrix weighted by edge degrees

(a) **(b1)**

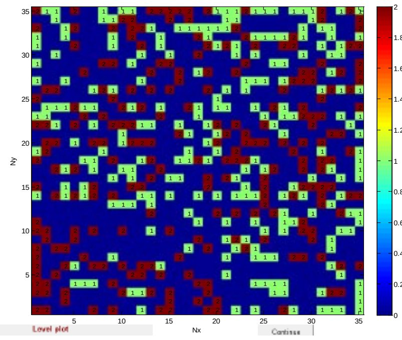

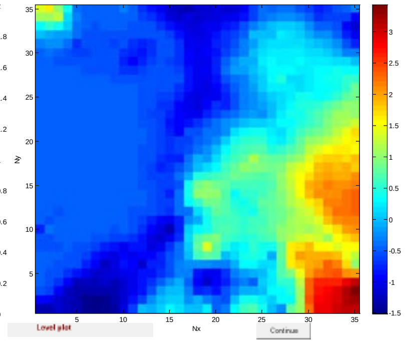

Position - n_x*n_y=35**2; 35*3; ; 35*4*

(a) ***Dragon descriptor D7***- *GGI2- Topological charge index of order 2* **(b2)**

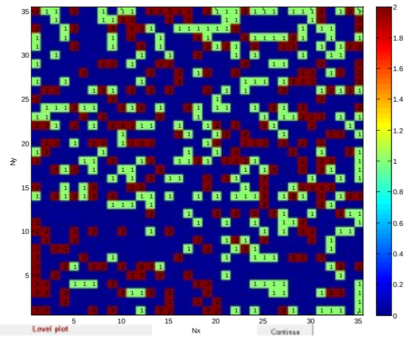

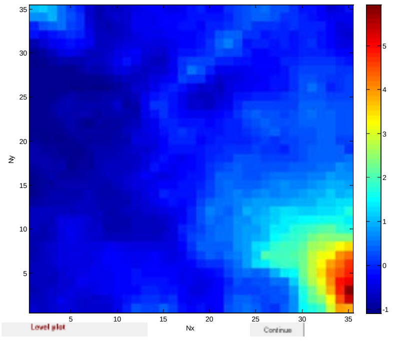

(c) Position - n_x*n_y=35**2; 35*3; ; 35*4*

*Position- n_x*n_y=35*2;* beta-Cyclodextrin (*CASRN 7585-39-9*); NA; NP in rat and mouse

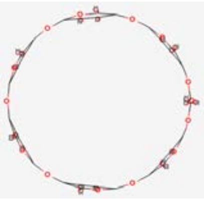

*Position - n_x*n_y=35*3;* Vinblastine; (*CASRN 865-21-4*); NA; NP

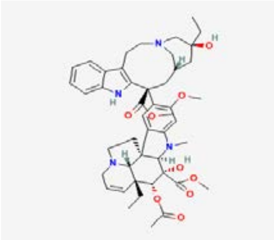

*Position - n_x*n_y=35*3;* Actinomycin D; (*CASRN 50-76-0*); GA; P

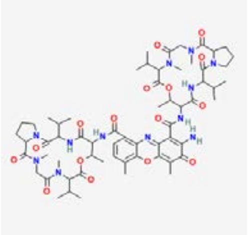

*Position - n_x*n_y=35*4;* Rifampicin; (*CASRN 13292-46-1*); SA13; NP

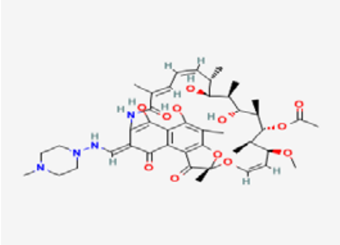

SA13- Hydrazine

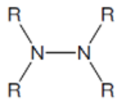

Figure S4. (**a**)- The output layer of model_cancer_***class*** with distribution of carcinogens (**2**) and non-carcinogens (**1**); (**b1, b2**)- The weight maps corresponding to descriptors ***D4*** and ***D7*** correspondingly; (**c**)- The name and structure of chemicals placed in the influential zone of descriptors ***D4*** and ***D7*** with indication of position in Kohonen map.

Dragon descriptor D5- *ESpm11x*- Spectral moment 11 from edge adj. matrix weighted by edge degrees

(a) **(b1)**

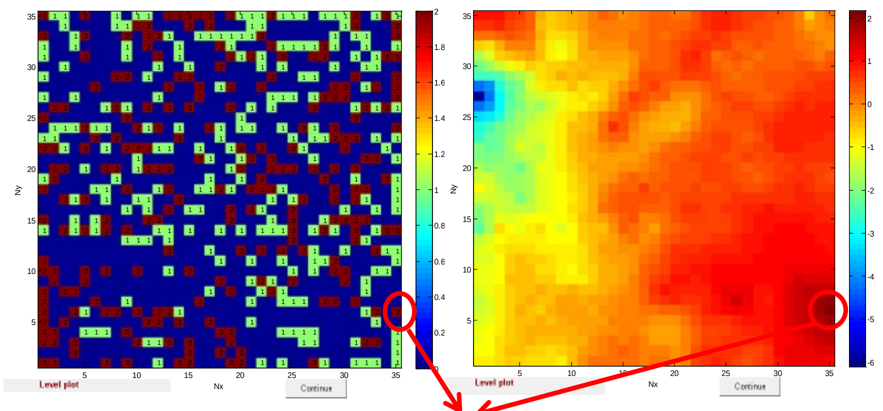

Position - $n_x * n_y = 35*6$

Dragon descriptor D6- *ESpm09d*- Spectral moment 09 from edge adj. matrix weighted by dipole moments

(a) **(b2)**

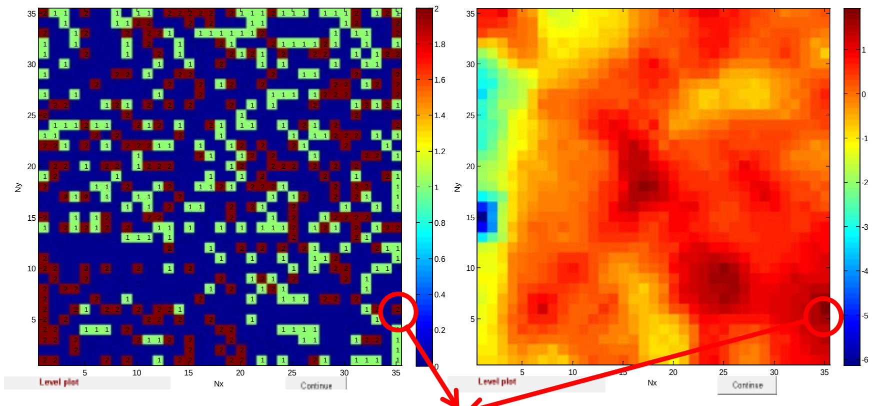

(c) Position - $n_x * n_y = 35*6$

Position of $n_x * n_y = 35*6$; Mirex, photo-; (*CASRN 39801-14-4*); SA20; P	Position of $n_x * n_y = 35*6$; Chlordecone (kepone); (*CASRN 143-50-0*); SA20; P
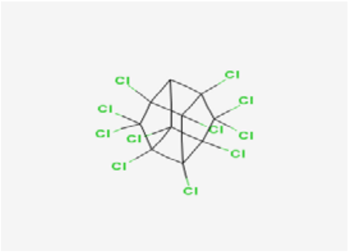	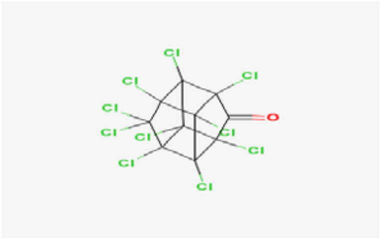

SA_20: (Poly) Halogenated Cycloalkanes (nongenotoxic)

Figure S5. (**a**)- The output layer of model_cancer_***class*** with distribution of carcinogens (**2**) and non-carcinogens (**1**); (**b1, b2**)- The weight maps corresponding to descriptors ***D5*** and ***D6***, respectively; (**c**)- The name and structure of chemicals placed in the influential zone of descriptors ***D5*** and ***D6*** with indication of position in Kohonen map.

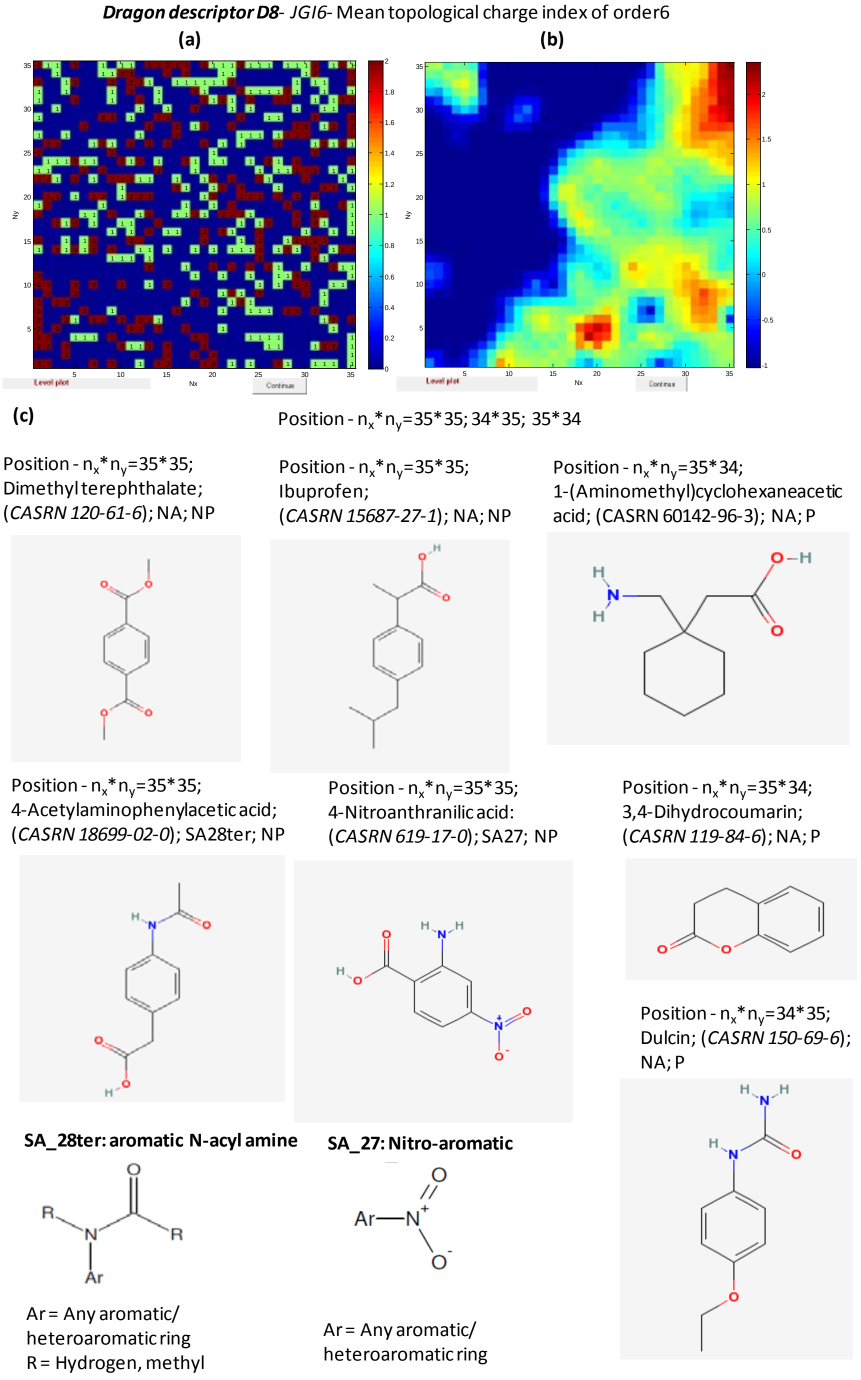

Figure S6. (**a**)- The output layer of model_cancer_***class*** with distribution of carcinogens (**2**) and non-carcinogens (**1**); (**b**)- The weight maps corresponding to descriptor ***D8*** (*JGI6*- Mean topological charge index of order6); (**c**)- The name and structure of chemicals placed in the influential zone of descriptor ***D8*** with indication of position in Kohonen map.

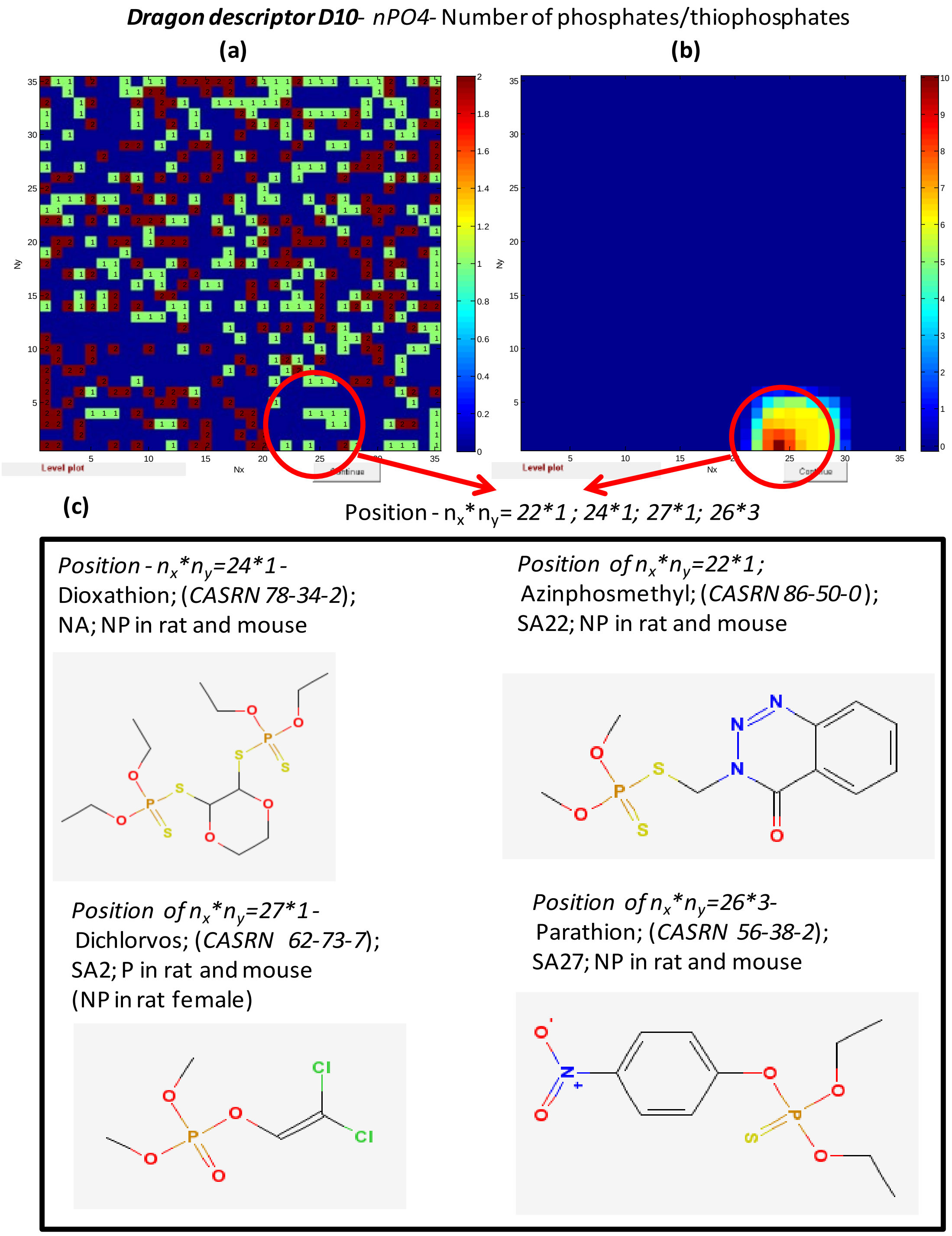

Figure S7. (**a**)- The output layer of model_cancer_***class*** with distribution of carcinogens (**2**) and non-carcinogens (**1**); (**b**)- The weight maps corresponding to descriptor ***D10 (*** *nPO4*- Number of phosphates/thiophosphates); (**c**)- The name and structure of chemicals placed in the influential zone of descriptor ***D10*** with indication of position in Kohonen map.

Dragon descriptor D11- *N-067*- Al2-NH

(a)

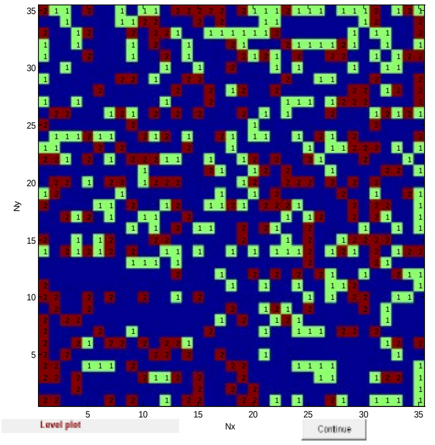

(b)

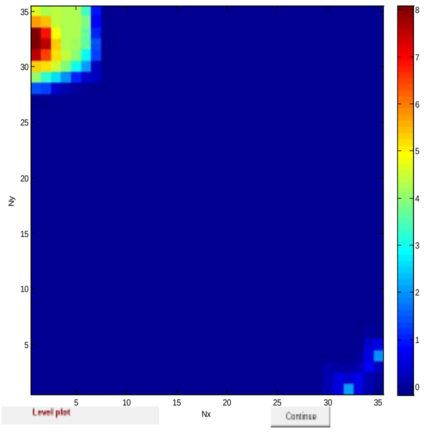

(c)

Position - n_x*n_y= 1*31; 1*32; 1*33

*Position - n_x*n_y=1*33;*
Cimetidine;
(*CASRN 51481-61-9*); NA; NP

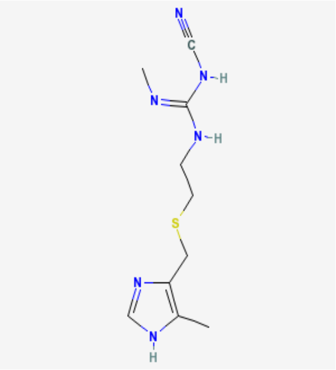

*Position - n_x*n_y=1*33;*
Procarbazine;
(*CASRN 671-16-9*); GA; P

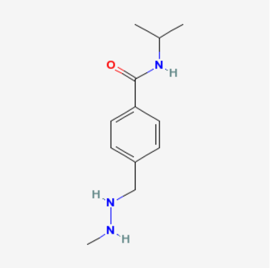

*Position - n_x*n_y=1*32;*
Mannitol nitrogen mustard;
(*CASRN 576-68-1*);
SA8; NP in rat, non avalable in mouse

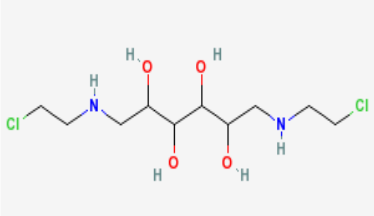

SA_8: Aliphatic halogens;
R = any atom/group

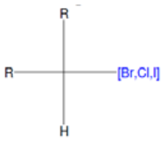

*Position - n_x*n_y=1*31;*
Piperazine; (*CASRN 110-85-0*); NA; NP

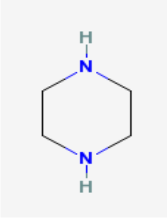

Figure S8. (**a**)- The output layer of model_cancer_***class*** with distribution of carcinogens (**2**) and non-carcinogens (**1**); (**b**)- The weight maps corresponding to descriptor ***D11*** (*N-067- Al2-NH*) ; (**c**)- The name and structure of chemicals placed in the influential zone of descriptor ***D11*** with indication of position in Kohonen map.

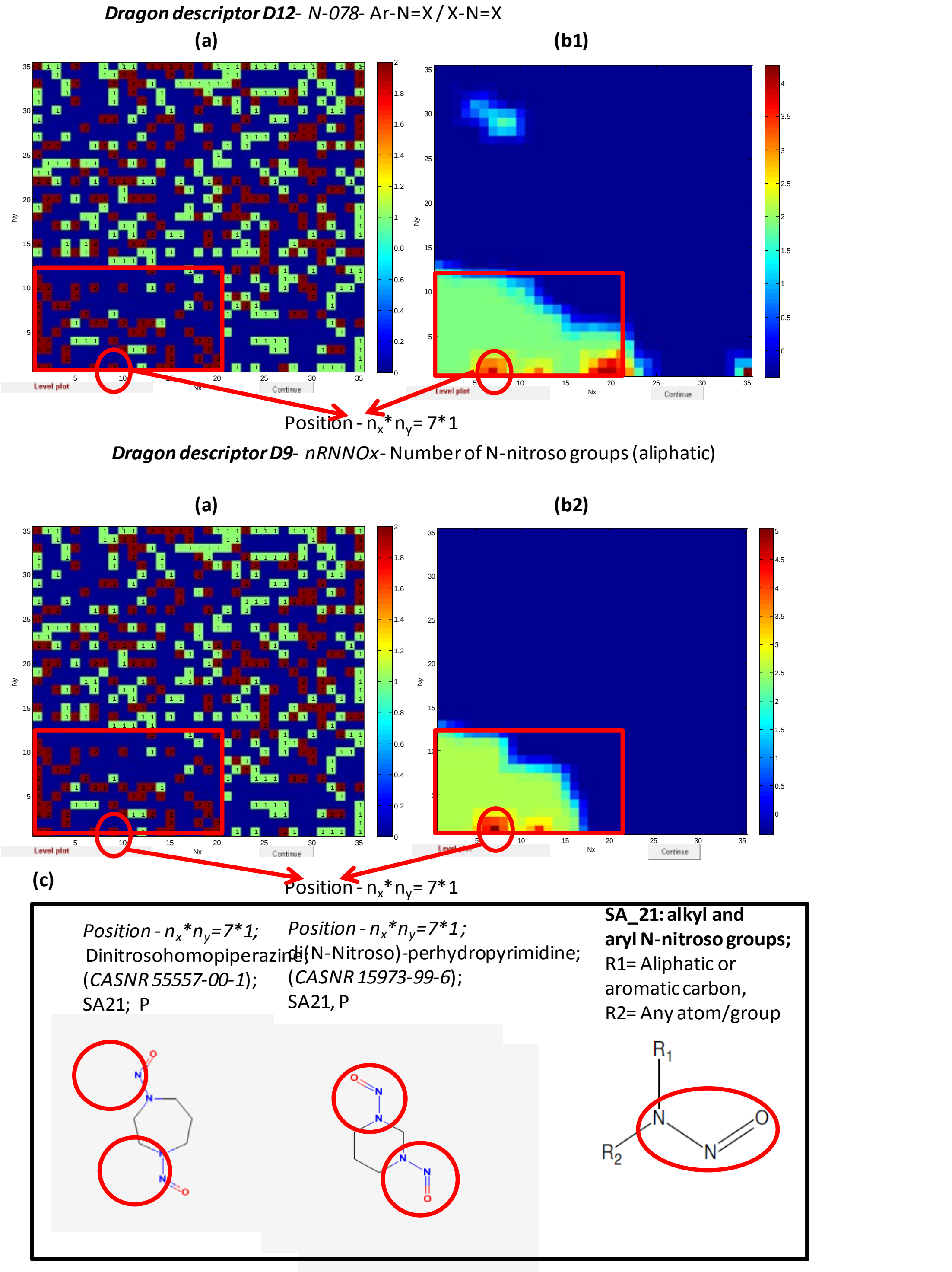

Figure S9. (**a**)- The output layer of model_cancer_***class*** with distribution of carcinogens (**2**) and non-carcinogens (**1**); (**b1, b2**)- The weight maps corresponding to descriptors ***D12*** and ***D9***, respectively; (**c**)- The name and structure of chemicals placed in the influential zone of descriptors ***D12*** and ***D9*** with indication of position in Kohonen map.

Notes:* Abbreviations in **Figures 1SI-9SI: GA- genotoxic alert; NA- no alert; P- positive (carcinogen); NP- non positive (non-carcinogen).

Table S1 The diversity of dataset used in CP ANN model with indication of number of carcinogens (P) and non-carcinogens (NP) as well as number of chemicals without carcinogenic alert (NA), genotoxic alerts (GA) and non-genotoxic alert (nGA)

**Alert/NA**	**P**	**NP**	**ALL**
**NA**	85	182	267
**GA**	225	110	335
**nGA**	19	15	34
**GA+nGA**	3	5	8
**Sum**	332	312	644

Table S2 Chemical structure of compounds contating (*SA13 + SA27*) (12 compounds)

ID_CPDBAS	CASRN	Structure	P/NP
8	18523-69-8	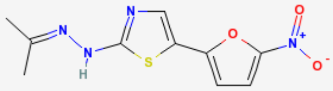	P
518	26049-69-4	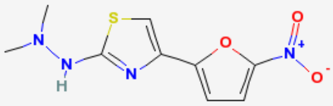	P
640	3570-75-0	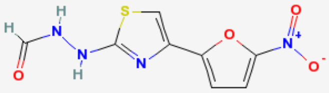	P
705	26049-68-3	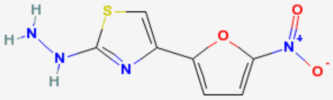	P

706	26049-70-7	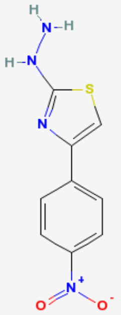	P
724	5036-03-3	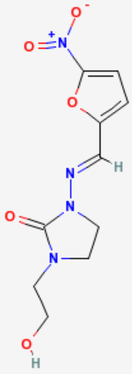	P
848	21638-36-8	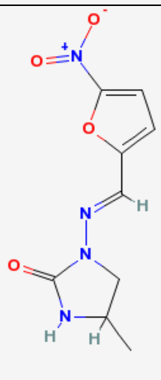	P
944	59-87-0	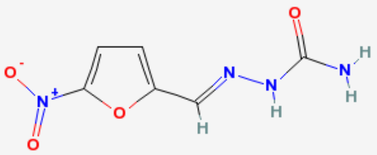	P

972	67-20-9	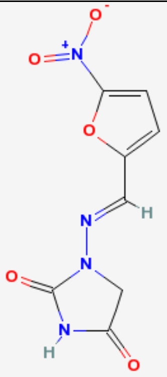	P
973	555-84-0	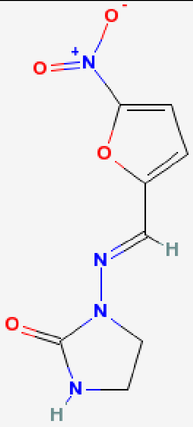	P
1226	2425-85-6	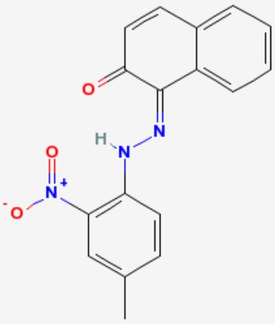	P
1227	6471-49-4	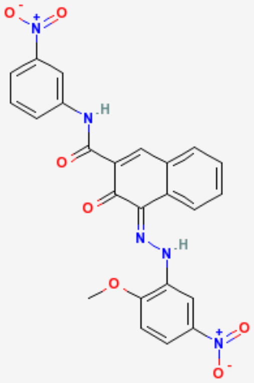	NP

Table S3 Chemical structure of compounds contating (*SA27_SA28*) (12 compounds)

ID_CPDBAS	CASRN	Structure	P/NP
58	3775-55-1	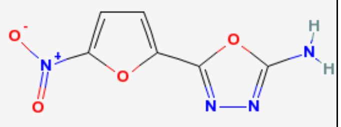	P
59	712-68-5	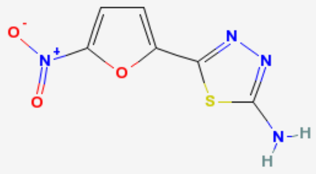	P
60	38514-71-5	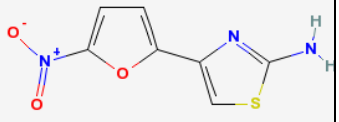	P
62	99-57-0	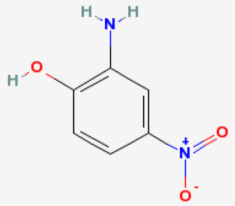	P

63	121-88-0	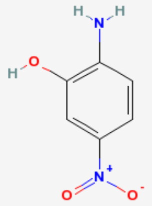	P
64	119-34-6	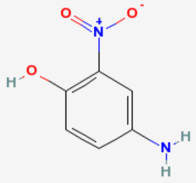	P
66	121-66-4	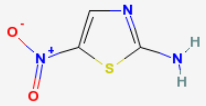	P
396	720-69-4	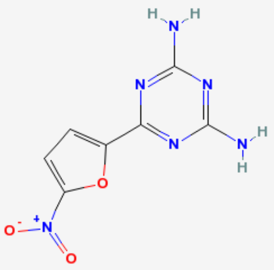	P
943	99-59-2	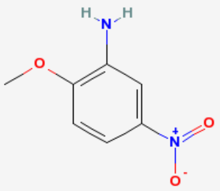	P

957	5307-14-2	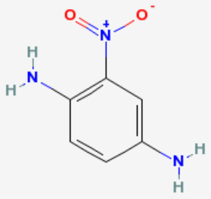	NP
958	99-56-9	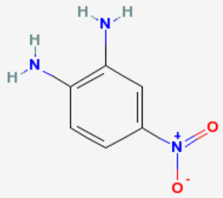	NP
959	99-55-8	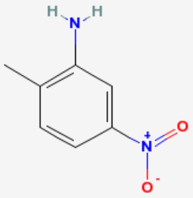	NP
961	100-01-6	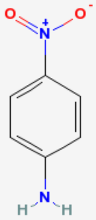	NP
1236	2871-01-4	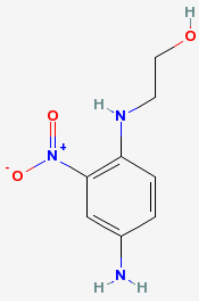	NP

Table S4 Chemical structure of compounds containing *SA_28* (52 compounds)

ID_CPDBAS	CASRN	Structure	P/NP
76	61-82-5	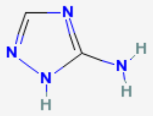	P
90	62-53-3	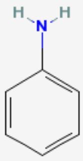	NP
137	92-87-5	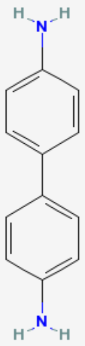	P
142	91-76-9	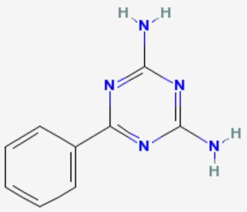	NP

262	133-90-4	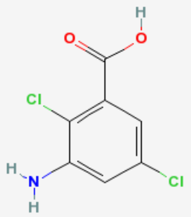	NP
275	101-79-1	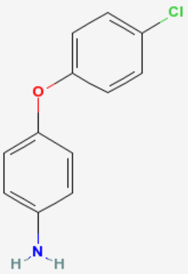	P
282	5131-60-2	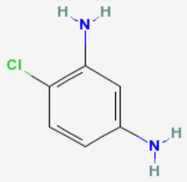	P
283	95-83-0	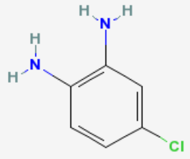	P
286	95-74-9	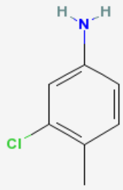	NP

287	95-79-4	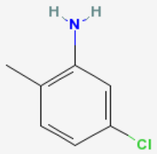	NP
295	106-47-8	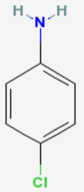	NP
349	102-50-1	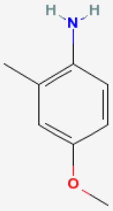	P
350	120-71-8	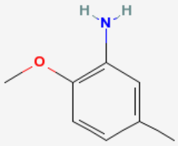	P
371	80-08-0	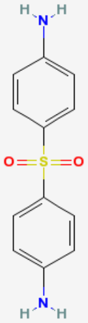	P

402	95-80-7	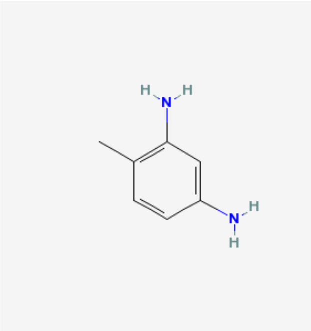	P
427	609-20-1	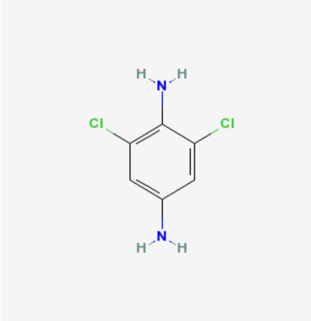	NP
432	91-94-1	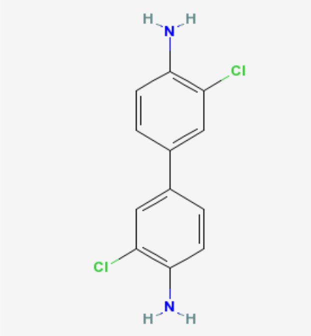	P
482	5803-51-0	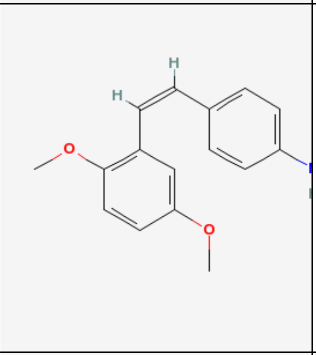	P
657	67730-11-4	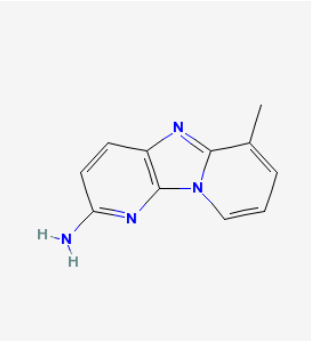	P

658	67730-10-3	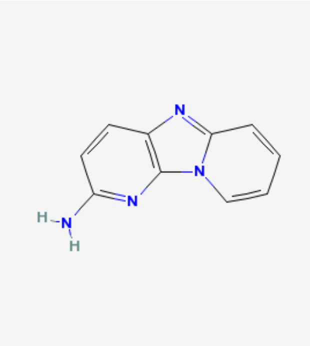	P
718	4463-22-3	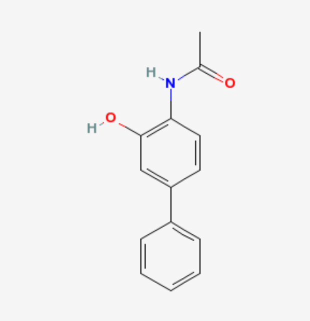	NP
720	4363-03-5	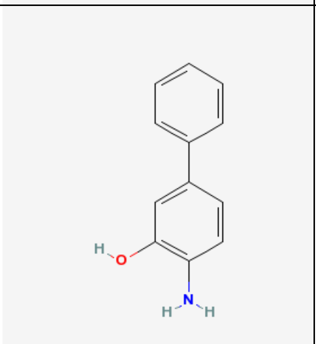	NP
802	108-78-1	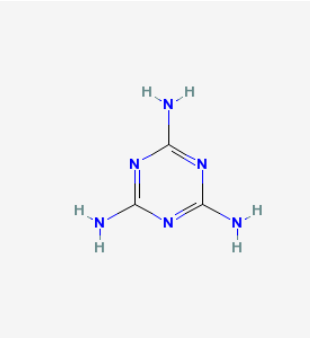	P
822	59-05-2	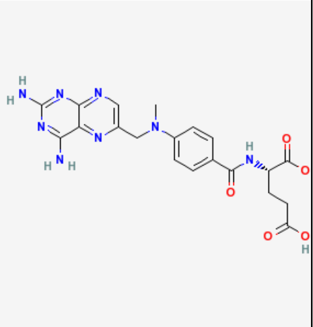	NP

865	101-14-4		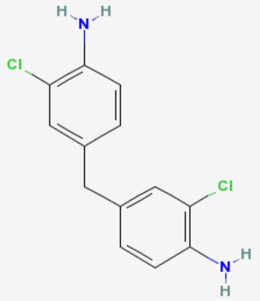	P
867	838-88-0		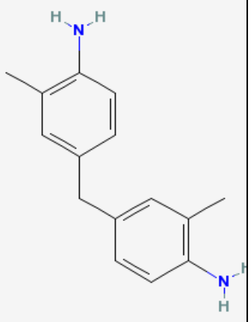	P
916	2243-62-1		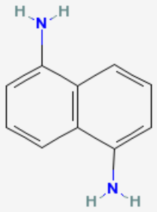	P
921	91-59-8		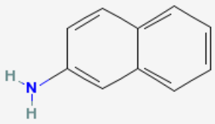	P
1094	101-80-4		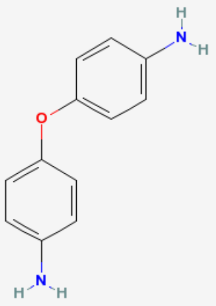	P

1138	106-50-3		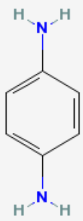	NP
1292	127-69-5		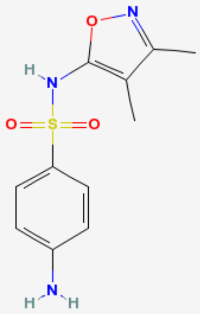	NP
1314	15721-02-5		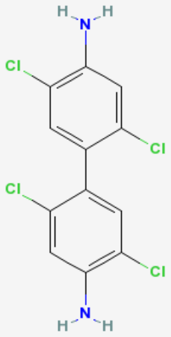	NP
1344	139-65-1		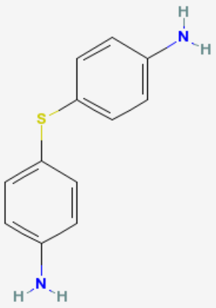	P
1373	396-01-0		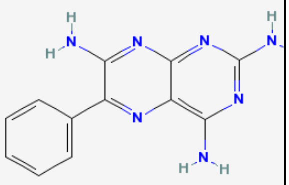	NP

1398	137-17-7	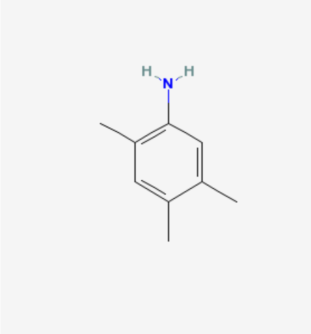	P
